# Ultrasonic AccV: a potential indicator of peripheral arteriosclerosis in patients with chronic obstructive pulmonary disease

**DOI:** 10.1186/s12890-024-02879-0

**Published:** 2024-02-09

**Authors:** Li Lin, Yuting Yan, Bin Jiang, Gang Hou, Yan Yin, Lei Wang, Jian Kang, Qiuyue Wang

**Affiliations:** 1 Department of Pulmonary and Critical Care Medicine, Institute of Respiratory Disease, The First Hospital of China Medical University, No. 155 Nanjing North Street, Shenyang, 110001 China; 2 Department of Critical Care Medicine, The affiliated hospital of Qingdao university, Qingdao, China; 3 Department of Ultrasound, The First Hospital of China Medical University, Shenyang, 110001 China; 4 Department of Pulmonary and Critical Care Medicine, China–Japan Friendship Hospital, Beijing, China; 5 Department of vascular surgery, The First Hospital of China Medical University, Shenyang, China

**Keywords:** Peripheral arteriosclerosis, Peripheral artery disease, Ankle-brachial index, Pulse wave velocity, Blood flow acceleration velocity, Peak systolic blood flow velocity

## Abstract

**Objective:**

This study aimed to investigate the risk factors for peripheral arteriosclerosis (PAS) and peripheral artery disease (PAD) in chronic obstructive pulmonary disease (COPD) patients and potential ultrasound indicators that could be used to improve detection.

**Method:**

Outpatients seeking care between January 1, 2017, and December 31, 2020, in The First Affiliated Hospital of China Medical University were prospectively recruited. Subjects were divided into COPD and non-COPD (control) groups, and the COPD group was further divided into PAD and non-PAD subgroup, at the same time, PAS and non-PAS subgroup. Indicators of PAD -ankle-brachial index (ABI), indicators of PAS- pulse wave velocity (PWV), and ultrasound indices -peak systolic blood flow velocity (PSV) and blood flow acceleration velocity (AccV) were compared.

**Result:**

Sixty-nine (61.6%) of 112 enrolled subjects had COPD. COPD patients had higher age, and blood pressure (BP)lower than controls. Seventeen (24.6%) COPD patients had PAD, the prevalence of PAD increases with the decrease of lung function, and seven (16.3%) non-COPD patients had PAD, however, there was no significant statistical difference between COPD and non-COPD groups. Fifty (72.5%) COPD patients had PAS, and thirty-four (79.1%) non-COPD patients had PAS, however, there was also no significant difference. The PAS subgroup had higher age, body mass index(BMI), body fat percentage(BFP), lower FEV1 and FEV1/FVC, as well as higher levels of right brachial artery and left dorsalis pedis artery AccV. Factors that correlated with ABI were 6MWD, post-bronchodilator FEV1, FEV1/ FVC, and maximal middle expiratory flow between 75% and 25% of FVC. Age, BP, and 6MWD, but not pulmonary function, were associated with brachial-ankle PWV (baPWV). There was a positive correlation between baPWV and radial artery AccV bilaterally.

**Conclusion:**

Radial artery AccV correlated well with baPWV, which suggests that ultrasound could be used to assess both morphological and functional changes in vessels, may serving as a better method to identify PAS in high-risk COPD patients.

## Introduction

Chronic obstructive pulmonary disease (COPD) is a common respiratory disease characterized by persistent respiratory symptoms and airflow limitations. The prevalence and mortality rates of COPD are rising rapidly both in China and globally, and the latest statistics indicate that the prevalence of COPD in people aged 40 years or older is 13.7% in China [1] and 8.9% in the USA [2]. The World Health Organization (WHO) estimates that COPD may become the third leading cause of death worldwide, imposing huge social and economic burdens on individuals, families, and society [3, 4]. Patients with COPD often suffer from comorbidities; studies have shown that up to 98% of COPD patients show one or more comorbidities [5], which affect patient’s prognosis to some extent. Understanding COPD as a complex multisystem disease is therefore crucial for its management.

Peripheral artery disease (PAD) is a form of peripheral arteriosclerosis (PAS), where narrowing and obstruction of the arteries by plaques impair blood flow and limit the supply of oxygen to tissues [6]. Broadly defined, PAD includes all atherosclerotic artery diseases other than cardiovascular or cerebrovascular disease, which is how PAS is defined in this study. However, in an everyday clinical context, PAD usually refers specifically to atherosclerotic occlusion of arteries in the lower extremities, also known as intermittent claudication syndrome, which is the definition of PAD used in the present study. PAS has a high prevalence rate and is often described as a ‘silent killer’ because a high number of individuals with PAS are asymptomatic and unaware of the disorder [7].

Cardiovascular disease (CVD) is the second leading cause of morbidity and mortality in COPD patients [8]. PAS are strongly associated with CVD. Similar to coronary heart disease (CHD), PAS increases the risk of major cardiovascular events, such as myocardial infarction and death [9; 10]. It was reported that PAS increases the relative risk of cardiovascular events and mortality in COPD patients by 14% and 15%, respectively [11]. Both COPD and PAS can be caused by cigarette smoking and the two conditions therefore often co-exist [6]. Patients with COPD are at increased risk of developing PAS and PAD and suffer more from PAS- and PAD-related disability and mortality [4; 10; 12; 13]. However, the burden of PAS and PAD on COPD patients, and vice versa, is not well-defined, especially in Asian populations [10]. Furthermore, few studies have evaluated other methods that might be more suitable for PAD and PAS screening than ABI and PWV in COPD patients.

The 2013 ACCF/AHA guidelines for the management of patients with PAD recommend the measurement of resting ankle-brachial index (ABI) for early screening and diagnosis of PAD [12]. ABI is defined as the ratio between systolic BP measured at the ankle to that measured at the brachial artery. PAD is a manifestation of atherosclerosis in the lower limbs, however, ABI only reflects the degree of stenosis of the arterial lumen, and not the actual degree of arteriosclerosis [14, 15], and thus has limitations when used to diagnose PAD in high-risk COPD patients.

Pulse wave velocity (PWV) is the most widely used indicator of arterial stiffness. PWV is calculated as the distance traveled by the pulse wave divided by the time taken to travel the distance. It is easy to operate, non-invasive, and has high repeatability combined with a low cost, which makes it an optimal method for screening and early diagnosis of PAS [16, 17, 18]. However, it only reflects blood vessel elasticity and not morphological changes.

Ultrasound is a simple non-invasive method that can be used to evaluate morphologic changes in arteries. Ultrasound can also be used to assess PAS and PAD by evaluating functional parameters. To the best of our knowledge, we are the first to evaluate the use of peak systolic blood flow velocity (PSV) and blood flow acceleration velocity (AccV) of the radial artery to assess PAS and PAD in patients with high-risk COPD. AccV reflects arterial acceleration, which could not only better reflect the arterial blood flow, but also reflect the arterial elasticity.

Knowledge about how to objectively assess PAS and PAD in patients with COPD is scarce. The present study investigated the prevalence of PAS and PAD in COPD patients in China using objective parameters including ABI, toe-brachial index (TBI), PWV, and ultrasound indicators to determine candidate methods for assessing PAS and PAD in COPD patients.

## Methodology

### Research subjects

This prospective study was conducted on outpatients seeking care between January 1, 2017, and December 31, 2020, in The First Affiliated Hospital of China Medical University. Patients were clinically diagnosed with COPD according to the GOLD 2017 guidelines. Patients without COPD were selected as the control group.

Diagnosis of PAD was based on the 2013 ACCF/AHA guidelines for the management of patients with PAD [19]. PAD was considered present when the ABI ≤ 0.9 or TBI ≤ 0.7 on either side and/or peripheral vascular lesions were detected using imaging such as Doppler ultrasound, digital subtraction angiography, computed tomography (CT) angiography, or magnetic resonance angiography.

BaPMV ≥ 1,400 cm/s was used as the criteria for diagnosis of PAS [20, 21, 22]. Were further divided into PAS and non-PAS subgroups.

### Inclusion criteria

1. Age 40 to 80 years;
2. Do not meet exclusion criteria;
3. Can communicate verbally or in writing;
4. Understand the information given and have signed the informed consent.

### Exclusion criteria

1. Presence of other respiratory diseases including asthma, bronchiectasis, active tuberculosis, diffuse panbronchiolitis, interstitial pulmonary diseases such as pneumoconiosis, alveolar proteinosis, idiopathic pulmonary fibrosis; and other diseases that cause airflow limitations;
2. Systemic disease comorbidity, including severe liver and kidney dysfunction, autoimmune disease, and severe cardiovascular and cerebrovascular disease;
3. Pregnant or lactating;
4. Patients who cannot complete vascular-related examinations due to severe arrhythmias;
5. Patients with severe disease, unable to cooperate with lung function, and related tests.

### Research methods

#### General information collection and questionnaire survey

Demographic and clinical information of the participants was collected in the questionnaire, including age, gender, height, weight, body mass index (BMI), body fat percentage (BFP), blood pressure (BP), peripheral oxygen saturation, smoking history, previous disease history, and years of COPD diagnosis. The Modified Medical Research Council COPD Assessment Test (CAT) and Clinical COPD Questionnaire (CCQ) scores were also included in this questionnaire.

#### Lung function measurement

All lung function measurements were carried out using a Jaeger Masterscreen lung function instrument (Jaeger Company, Germany) operated by a dedicated and experienced person Quality control was performed in accordance with the American Thoracic Society (ATS) standard for pulmonary function measurement [23].

#### Peripheral atherosclerosis detection

An arteriosclerosis detection device (BP-203RPEIII, Omron, Japan) was used to detect PAS in all patients. Parameters including bilateral brachial ankle-PWV (baPWV), bilateral ABI and TBI were measured. Measurements were conducted by a dedicated and experienced person. Quality control was performed in accordance with the 2016 Society of Interventional Radiology and the Society of Interventional Radiology of Canada’s position statement on non-invasive peripheral artery imaging [24].

#### PAS and PAD examination using ultrasound

Ultrasound was performed using an IU22 ultrasonic diagnostic instrument and L5-12 vascular probe and program (Philips, Dutch). The angle between the acoustic beam and blood flow was < 60° and the sampling volume was 1.5−2.0 mm. Participants were examined in a sitting position, with the upper limbs flexed and in abduction, knees bent, and feet flat on the examination table. Bilateral brachial artery (10 cm above the elbow fossa, where the artery is flat) and dorsalis pedis artery (2–3.5 cm below the line connecting medial and lateral malleolus, where the artery is straight) were shown, respectively. The PW program was chosen to obtain a complete arterial blood flow spectrum, capturing PSV and AccV. Each side was measured three times, and the average value was calculated.

#### Exercise endurance measurement

The 6-minute walk distance (6MWD) test was recorded according to the 2002 ATS guidelines. An indoor, 30 m long straight corridor was chosen. Investigators explained the method to the patients before the test and told them to walk as far as possible. Patients were instructed to slow down, stop, and rest if have symptoms such as shortness of breath, chest pain, or dizziness appeared. If the symptoms were not relieved by resting, the test was stopped immediately. The test was supervised by an investigator, and standard language was used to encourage patients. The patients stopped walking after hearing the stop signal of a timer and the walking distances were recorded.

### Statistical analysis

All statistical analyses were carried out using SPSS25.0 software. We used PASS software to calculate the sample size. Normally distributed data are expressed as mean ± standard deviation (x̅±SD) and compared using the student’s t-test. Non-normally distributed data are expressed as median and range and compared using non-parametric tests. Count data were compared using the Chi-square test. Multivariate analysis was performed using Pearson correlation analysis. A *p*-value < 0.05 was considered statistically significant.

## Results

### Prevalence of PAD and PAS in COPD

**Fig. 1:**
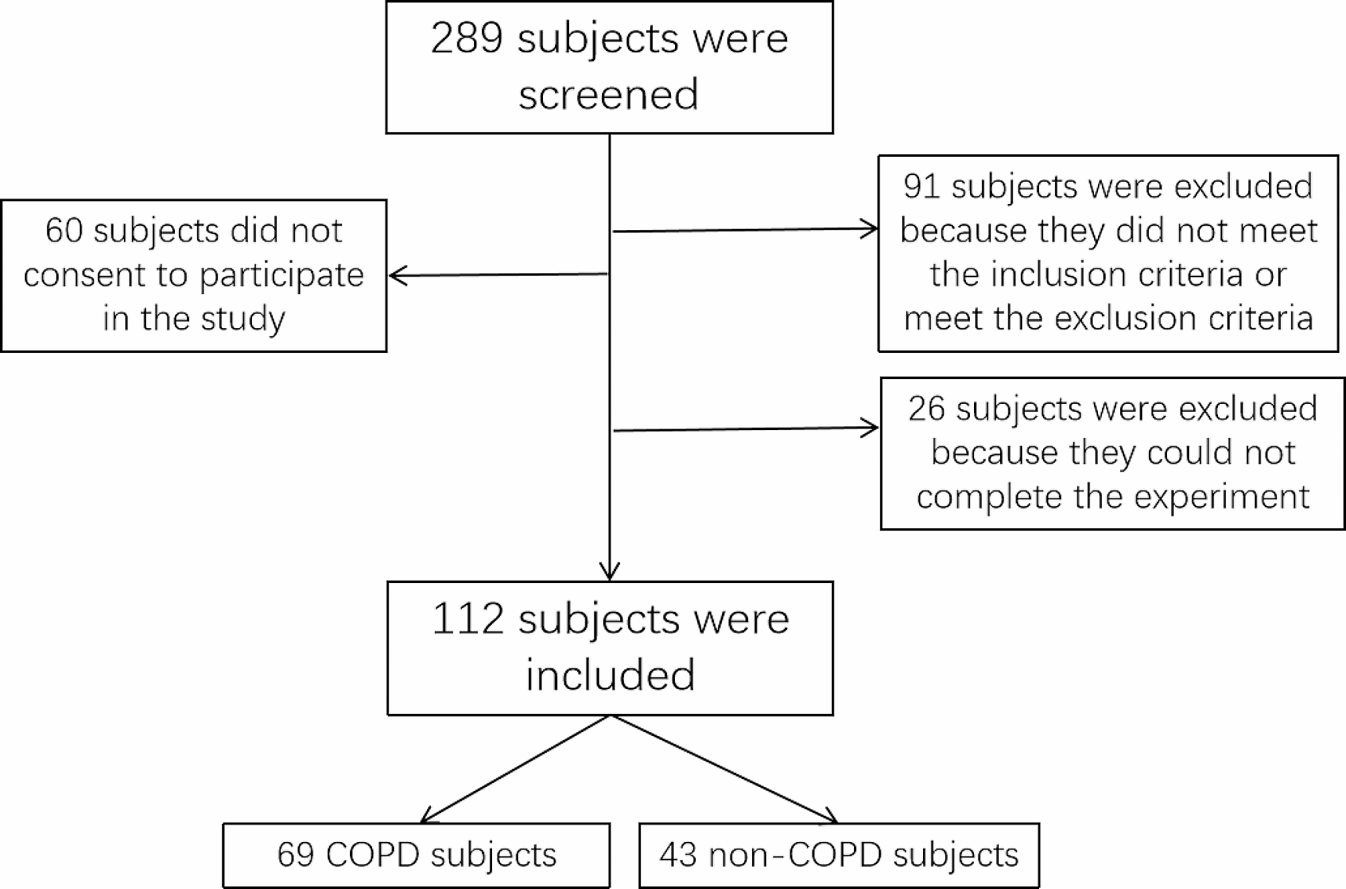
The flowchart of this study

A total of 289 subjects were screened for inclusion from January 1, 2017, to December 31, 2020, and 112 subjects who met the inclusion criteria were included in the study. Among these patients, 69 subjects were diagnosed with COPD according to the GOLD criteria and 43 subjects did not have COPD(Fig. 1). Most COPD patients had mild to moderate disease. 11 had GOLD stage I, 39 had GOLD stage II, 16 had GOLD stage III, and 3 had GOLD stage IV. In the COPD group, seventeen patients (24.6%) had PAD, including two patients (18.2%) in Grade I, eight patients (20.5%) with Grade II, four patients (25%) with Grade III, and three patients (100%) with Grade IV disease. Seven cases (16.3%) PAD were identified in the non-COPD group. But none of these patients had significant signs of Leriche’s disease. The risk factor PAD in COPD group is 0.246 (95% confidence interval 0.142–0.351). The incidence of PAD in COPD patients was higher than that in non-COPD patients, and the incidence of PAD further increased with the decline of lung function, according to statistics, the PAD morbidity is higher in Grade IV than in other grade groups and non-COPD group (Table 1). Unlike COSYCONET studies [25], this study did not find a statistical difference in PAD prevalence between COPD and non-COPD groups, possibly due to insufficient sample size in this study.

**Table 1:** The incidence of PAD was compared between COPD groups and non-COPD group

Variable	PAD	non-PAD	*n*	Morbidity(%)	χ2	*p*
**non-COPD group**	**7**	**36**	**43**	**16.3**		
**COPD Grade I**	**2**	**9**	**11**	**18.2**	**0.023**	**0.880** ^**#**^
**COPD Grade II**	**8**	**31**	**39**	**20.5**	**0.245**	**0.620** ^**#**^
**COPD Grade III**	**4**	**12**	**16**	**25**	**0.585**	**0.444** ^**#**^
**COPD Grade IV**	**3**	**0**	**3**	**100**	**11.553**	0.001*
**COPD group**	**17**	**52**	**69**	**24.6**	**1.099**	**0.294**

*compared with non-COPD group *p* < 0.05, # compared with COPD grade IV group *p* < 0.05

In the COPD group, fifty patients (72.5%) had PAS, including seven patients (63.6%) in Grade I, twenty-six patients (66.7%) with Grade II, fourteen patients (87.5%) with Grade III, and three patients (100%) with Grade IV disease. The risk factor PAS in COPD group is 0.725 (95% confidence interval 0.617–0.833). The incidence of PAS in COPD patients increased with the decrease of pulmonary function, but statistical analysis did not show significant difference. Thirty-four cases (79.1%) PAS were identified in the non-COPD group. There was no significant difference in the prevalence of PAS between COPD and non-COPD groups (Table 2).

**Table 2:** The incidence of PAS was compared between COPD groups and non-COPD group

Variable	PAS	non-PAS	*n*	Morbidity(%)	χ2	*p*
**non-COPD group**	**34**	**9**	**43**	**79.1**		
**COPD Grade I**	**7**	**4**	**11**	**63.6**	**1.141**	**0.285**
**COPD Grade II**	**26**	**13**	**39**	**66.7**	**1.603**	**0.206**
**COPD Grade III**	**14**	**2**	**16**	**87.5**	**0.546**	**0.460**
**COPD Grade IV**	**3**	**0**	**3**	**100**	**0.781**	**0.377**
**COPD group**	**50**	**19**	**69**	**72.5**	**0.617**	**0.432**

### Clinical data

The average age was higher and BMI was lower in the COPD group compared to the non-COPD group, which also suggests the occurrence of systemic complications of COPD. There were no statistically significant differences in BFP and the proportion of comorbidities (hypertension, diabetes, and CHD). Results are shown in Table 3.

**Table 3:** Comparison of general information between COPD and non-COPD groups

Variable	COPD (*n* = 69)	Non-COPD (*n* = 43)	*p* Value
**Gender (Male/Female)**	49/20	24/19	0.096
**Age (Year)**	65.5 ± 7.4	59.7 ± 10.5	0.001
**BMI (kg/m** ^**2**^ **)**	23.0 ± 3.7	24.4 ± 3.5	0.041
**Body fat (%)**	28.4 ± 7.3	29.2 ± 5.6	0.574
**Hypertension (With/Without)**	9/40	12/20	0.055
**Diabetes (With/Without)**	4/38	3/29	0.938
**CHD (With/Without)**	2/45	3/28	0.339

Definition of abbreviation: CHD: coronary heart disease

PAS was present in of the remaining 84 patients. The patients in the PAS group were older, and the BMI and BFR were higher, which is consistent with previous researches. There was no significant difference in the proportions of comorbidities between the PAS and non-PAS groups. (Table 4).

**Table 4:** Comparison of general information between PAS and non-PAS subgroup in COPD group

Variable	PAS (*n* = 84)	Non-PAS (*n* = 28)	*p* Value
**Gender (Male/Female)**	50/24	15/13	0.709
**Age (Year)**	66.7 ± 7.5	61.0 ± 5.6	0.001
**BMI (kg/m** ^**2**^ **)**	23.4 ± 3.7	21.3 ± 3.2	0.020
**Body fat (%)**	30.4 ± 6.5	22.6 ± 6.8	0.001
**Hypertension (With/Without)**	8/30	1/10	0.662
**Diabetes (With/Without)**	3/28	1/10	0.999
**CHD (With/Without)**	2/34	0/11	0.999

Definition of abbreviation: CHD: coronary heart disease

The COPD group had significantly higher cigarette years of smoking than the non-COPD group (*p* < 0.05). BP and 6MWD were both significanty lower in the COPD group (*p* < 0.05). No statistical differences in baPWV, ABI, and TBI were found between the two groups (*p* > 0.05). The forced expiratory volume in the first second (FEV1) and FEV1/forced vital capacity (FVC) were lower in the PAD group. These results suggest that the development of PAD is directly related to the decline of lung function in patients.(Table 5).

**Table 5:** Clinical Data in different groups

	COPD		non-COPD	
	non-PAD(*n* = 52)	PAD(*n* = 17)	non-PAD(*n* = 36)	PAD(*n* = 7)
**Nicotine usage, ** *n * **(%)**	30(69.8)	14(93.3)	10(62.5)	5(83.3)
Never	**13**	**1**	**6**	**1**
Current	**27**	**12**	**8**	**5**
Former	**3**	**2**	**2**	**0**
Missing	**9**	**2**	**10**	**1**
Number of cigarette years, mean ± SD (n)	**397.26 ± 442.86**	665.33 ± 356.74*	**283.31 ± 279.80**	**405 ± 305.53**
**Vital signs before 6 MW, mean ± SD**				
Resting state SBP(Millimetre of mercury)mean ± SD	**114.29 ± 15.93**	**118.53 ± 18.31**	**118.38 ± 30.48**	**127.25 ± 19.87**
Resting state DBP(Millimetre of mercury)mean ± SD	**73.77 ± 11.66**	**79.06 ± 14.74**	**76.64 ± 12.93**	**73 ± 3.92**
Oxygen saturation (%), mean ± SD	**95.37 ± 1.83**	**95.58 ± 1.44**	**95.79 ± 1.78**	**95.5 ± 1.52**
Heart Rate	**74.93 ± 10.43**	**80.85 ± 14.71**	**72.63 ± 10.15**	**80.17 ± 9.54**
**6MWD(m), mean ± SD**	**371.33 ± 62.44**	**351.05 ± 62.48**	**375.67 ± 59.89**	**370.83 ± 71.69**
**Vital signs after 6 MW, mean ± SD**				
Resting state SBP(mmHg), mean ± SD	**124.48 ± 14.39**	**129.50 ± 16.45**	**129.00 ± 13.33**	**134 ± 11.79**
Resting state DBP(mmHg), mean ± SD	**74.36 ± 9.02**	**78.30 ± 12.30**	**76.57 ± 9.31**	**73.67 ± 12.06**
Oxygen saturation (%), mean ± SD	**93.74 ± 3.31**	**93.67 ± 2.43**	**94.44 ± 2.44**	**94.5 ± 2.07**
Heart Rate	**86.10 ± 21.19**	98.42 ± 16.11*	**89.93 ± 15.31**	91.5 ± 11.18*
**Spirometry test, mean ± SD (n)**				
FVC(L)	**2.93 ± 0.97**	**2.75 ± 0.89**	**3.09 ± 0.93**	**3.09 ± 1.20**
FEV1 (L)	**1.71 ± 0.64**	**1.5 ± 0.72**	**1.83 ± 0.64**	**2.14 ± 0.93**
FEV1% predicted	**65.71 ± 18.38**	58.24 ± 27.28*	**77.95 ± 20.61**	**75.51 ± 16.30**
FEV1/FVC ratio (%)	**57.15 ± 7.41**	52.08 ± 11.75*	**89.85 ± 11.50**	73.52 ± 7.45*
PEF (L/min)	**4.61 ± 1.96**	**4.48 ± 1.74**	**4.59 ± 1.79**	**5.56 ± 2.99**
MMEF75/25	**0.77 ± 0.38**	**0.7 ± 0.46**	**1.00 ± 0.576**	**1.14 ± 0.72**
**CAT total score, mean ± SD**	**8.5 ± 4.23**	**8 ± 6**	**3.5 ± 7.78**	**5 ± 6.48**
**mMRC grade, mean ± SD**	**1.67 ± 0.69**	**0.5 ± 0.5**	**0.5 ± 0.71**	**0.5 ± 0.82**
**CCQ score, mean ± SD**	**7.33 ± 6.55**	**15 ± 10**	**5.5 ± 2.12**	**9 ± 2.12**
**PAD evaluation index**				
Left ABI	**1.13 ± 0.06**	**0.7 ± 0.1**	**1.13 ± 0.07**	**0.83 ± 0.15**
Right ABI	**1.16 ± 0.09**	**0.7 ± 0.11**	**1.16 ± 0.08**	**0.89 ± 0.10**
Left TBI	**0.87 ± 0.07**	**0.61 ± 0.08**	**0.91 ± 0.11**	**0.67 ± 0.20**
Right TBI	**0.82 ± 0.06**	**0.69 ± 0.19**	**0.84 ± 0.12**	**0.64 ± 0.10**
**PAS evaluation index**				
Left baPWV (cm/s)	**1333.48 ± 308.33**	**1680.41 ± 537.57**	**1311.89 ± 507.62**	**1543.29 ± 214.63**
Right baPWV (cm/s)	**1321.19 ± 293.30**	**1657.24 ± 495.33**	**1367.20 ± 411.85**	**1511.43 ± 219.74**

* Compared with the corresponding non-PAD group *p* < 0.05

### Correlation between clinical parameters and degree of atherosclerosis

Correlation analysis showed that left baPWV was positively correlated with age, BFP, CAT score, and BP (*p* < 0.05), but inversely correlated with 6MWD (*p* < 0.05); right baPWV was positively correlated with age and BP (*p* < 0.05), but inversely correlated with 6MWD (*p* < 0.05); these are consistent with the clinical features of PAS. No correlation was found between baPWV and BMI, cigarette years of smoking, mMRC, CCQ, or pulmonary function parameters (*p* > 0.05). Left ABI was positively correlated with 6MWD and certain pulmonary function parameters after inhalation of a bronchodilator, including FEV1, FEV1%, FVC, and maximal middle expiratory flow between 75% and 25% of FVC (MMEF75/25) (*p* < 0.05), but negatively correlated with CCQ (*p* < 0.05). Right ABI was positively correlated with 6MWD and certain pulmonary function parameters including FEV1, FEV1/ FVC, and MMEF75/25 post-bronchodilatation. These results suggest that the development of PAD is directly related to the decline of lung function in patients. ABI did not correlate with age, BMI, BFP, CAT, mMRC, BP, or cigarette years of smoking. The results are shown in Table 6.

**Table 6:** Correlation between bilateral baPWV, ABI, and other clinical parameters

		Age	BMI	BFP	Smoking	CAT Score	mMRC Score	CCQ Score	SBP	DBP	6MWD	FEV1	FEV1/ pred%	FEV1/FVC	MMEF75/25
**Left baPWV**	*r*	0.446	0.152	0.216	0.037	0.265	0.059	0.193	0.417	0.370	-0.264	-0.132	-0.056	-0.124	-0.14
	*p*	0.000	0.087	0.041	0.712	0.028	0.630	0.228	0.000	0.001	0.009	0.151	0.544	0.180	0.129
**Right baPWV**	*r*	0.446	0.124	0.186	0.119	0.236	0.001	0.073	0.378	0.354	-0.252	-0.161	-0.096	-0.161	-0.162
	*p*	0.000	0.162	0.078	0.237	0.051	0.996	0.649	0.000	0.001	0.013	0.079	0.298	0.078	0.078
**Left ABI**	*r*	-0.020	-0.125	-0.036	-0.124	-0.221	-0.194	-0.345	-0.086	-0.066	0.224	0.241	0.232	0.300	0.235
	*p*	0.820	0.162	0.740	0.220	0.071	0.112	0.029	0.439	0.522	0.028	0.008	0.011	0.001	0.01
**Right ABI**	*r*	-0.066	-0.138	-0.020	-0.148	-0.138	-0.166	-0.172	-0.030	-0.019	0.235	0.197	0.160	0.265	0.184
	*p*	0.460	0.121	0.853	0.142	0.257	0.174	0.283	0.785	0.866	0.021	0.031	0.081	0.003	0.045

Definition of abbreviation: baPWV: brachial-ankle pulse wave velocity; ABI: ankle brachial index; BMI: body mass index; BFP: body fat percentage; CAT: COPD assessment test; mMRC: modified Medical Research Council; CCQ: clinical COPD questionnaire; SBP: systolic blood pressure; DBP: diastolic blood pressure; 6MWD: 6-min walk distance; FEV1: forced expiratory volume in the first second; FEV1/pred%: FEV1% of predicted; FEV1/FVC: forced expiratory volume in the first second/forced vital capacity; MMEF75/25: maximal middle expiratory flow between 75% and 25% of FVC.

### Evaluating ultrasound indicators

Detection of PAS using ultrasound found that the right brachial artery AccV and left dorsalis pedis artery AccV were significantly correlated with PAS occurrence in COPD patients in this study. Correlation analysis showed that AccV was positively correlated with baPWV in the bilateral brachial artery and left dorsalis pedis artery (*p* < 0.05). These results suggests that AccV is significantly correlated with PAS, and AccV may be a potential indicator for assessing PAS. No correlation was found between PeakV and baPWV or ABI and ultrasound indices. The results are shown in Table 7.

**Table 7:** Correlation analysis of bilateral baPWV, ABI, and arterial ultrasound indices

			Left brachial artery PeakV	Left brachial artery AccV	Right brachial artery PeakV	Right brachial artery AccV	Left dorsalis pedis artery PeakV	Left dorsalis pedis artery AccV	Right dorsalis pedis artery PeakV	Right dorsalis pedis artery AccV
**Left baPWV**		*r*	0.110	0.313	0.072	0.339	0.106	0.202	-0.057	0.144
		*p*	0.239	0.001	0.444	0.000	0.264	0.032	0.547	0.124
**Right baPWV**		*r*	0.134	0.321	0.051	0.335	0.089	0.194	-0.063	0.121
		*p*	0.152	0.000	0.587	0.000	0.349	0.040	0.501	0.195
**Left ABI**		*r*	0.068	0.153	0.096	0.108	-0.067	0.030	-0.051	0.132
		*p*	0.468	0.102	0.310	0.251	0.482	0.756	0.590	0.159
**Right ABI**		*r*	0.032	0.080	0.124	0.079	0.056	0.062	0.063	0.117
		*p*	0.372	0.391	0.185	0.399	0.556	0.517	0.499	0.21

Definition of abbreviation: baPWV: brachial-ankle pulse wave velocity; ABI: ankle brachial index; PeakV: peak systolic blood flow velocity; AccV: blood flow acceleration

## Discussion

Individuals with COPD have an almost doubled risk of developing PAD. People suffering from both diseases have substantially higher mortality rates than those with COPD or PAD alone. Emphysema severity is associated with arterial stiffness in patients with COPD, but the pathophysiological mechanisms behind this association is not fully understood. Studies have shown that arterial elasticity and endothelial function are significantly lower in patients with COPD compared to healthy controls, regardless of the presence of PAS. The higher prevalence of PAS among patients with COPD than those without might be due to common etiological factors underlying both COPD and PAS, such as old age, male gender, long-term smoking, comorbidity with diabetes mellitus, and a chronic inflammatory state. BMI of COPD patients has been shown to decrease, while BMI of patients with PAD increases, which is often masked by the the combination of the two. Therefore, the disease is somewhat silent, which increases the need for more sensitive and specific methods to screen for PAD in COPD patients.

Multiple studies have revealed that decreased pulmonary function in COPD is closely related to PAS. The associations were independent of smoking and other major causes of endothelial dysfunction. Some researchers even proposed that airflow limitation and endothelial dysfunction are unrelated and independent predictors of PAS. Gene ontology enrichment analysis demonstrated that the up-regulated hub genes were mainly involved in the inflammatory response, reactive oxygen species metabolic process, cell adhesion, lipid metabolic process, regulation of angiogenesis, eicosanoid biosynthetic process, and cellular response to a chemical stimulus. The immune response may also be linked to the development and progression of COPD and PAS [26].

The overlap between COPD and PAS poses a diagnostic challenge and seriously affects the quality of life and prognosis of patients. There was lack of ultrasound parameters to assess PAS in addition to evaluating morphological changes, especially in high-risk COPD subjects. To our knowledge, the present study is the first to use AccV and PSV to evaluate PAS.

The current study included 128 subjects (85 COPD outpatients, and 43 non-COPD outpatients). The prevalence of PAD was relatively low, but still more than twice as high in the COPD group (5.9%) compared to the non-COPD group (2.3%). In Scotland, it was reported that the prevalence of PAD was 3.2% among 15,737 subjects aged 30 to 75 years [27]. In a cross-sectional study in Colombia, the prevalence of PAD in subjects over the age of 40 was 4.4% [28]. A recent large clinical study involving 2,088 patients with COPD in Germany reported that the prevalence of PAD among COPD patients was 8.8% [29], which is much higher than the prevalence of PAD among COPD patients in the present study. The prevalence of PAD in the present study was lower than that reported in both the COPD and the non-COPD groups. This could be due to a lower prevalence of PAD in China, which was reported in a previous study [30]. Other possible reasons for the lower prevalence might be the small sample size of the present study, or that only patients with mild disease were included, since we only included outpatients and the majority were stage I and II. This could also be attributed to using ABI to screen for PAD in COPD, which might have caused us to overlook some patients with early PAD.

Due to the low incidence of PAD in this study, we also analyzed the incidence and related factors of PAS in COPD patients. COPD and PAS patients share similar risk factors which makes it difficult to clinically differentiate between these two conditions. PAS was present in 77.4% of the COPD patients in the current study. Compared with the non-PAS group, the patients in the PAS group were older and their BMI and BFR were higher. The FEV1 and FVC (post-bronchodilator) in the PAS group were lower than in the non-PAS group, which is consistent with previous reports [34]. COPD and PAS share the common higher related factors - higher age and heavy smoke. Therefore, we should pay more attention to the combination of PAS in elderly patients with COPD.

COPD patients in this study had relatively higher age and smoking, lower BP, BMI, and 6MWD compared to patients in the non-COPD group. Low BP may be due to the high pulmonary hypertension in COPD patients, which affects left heart preload and may mask the effect of PAS on BP in COPD patients. Unfortunately, we did not evaluate the cardiac function or pulmonary artery pressure.The present study found no statistical differences in baPWV, ABI, and TBI between the COPD and non-COPD groups. There is significant higher tobacco use (pack- year) in COPD group than control group. But baPWV and ABI are not correlated with the result. We consider that this may be due to our limited sample size and the generally mild disease of the patients we observed.

ABI is commonly used to screen PAD, but the sensitivity and specificity are limited. The present study showed that ABI covaries with pulmonary function parameters [31; 32]. Therefore, using ABI to diagnose PAD in COPD might lead to overdiagnosis. In addition, ABI only reflects the degree of stenosis of the arterial lumen and does not take blood vessel elasticity into account [33], which leads to some sensitivity and specificity limitations of using ABI to diagnose PAD in high-risk COPD patients [34]. First, changes in arterial stiffness (elasticity) precede structural changes [35]. Therefore, screening for PAD using ABI may fail in the early diagnosis of PAD in high-risk groups. Secondly, ABI measurements are affected by the examiner; different examiners can obtain different results. Thirdly, when ABI is at a critical value (0.9−1.0), further tests will be needed to confirm the diagnosis [36]. Fourthly, the specificity of ABI in the diagnosis of PAD in a high-risk patient group is poor [12]. If latent lower extremity arterial disease (LEAD) is suspected, normal ABI (> 0.9) does not completely rule out LEAD, and further diagnosis by ABI after exercise or B ultrasound is required [37]. Finally, patients with diabetes or end-stage chronic kidney disease can have severe calcification in the middle artery, which is prone to falsely increasing ABI [38].

Correlation analysis showed that baPWV, an indicator of PAS, was positively correlated with age, but inversely correlated with exercise endurance as measured by the 6MWD test and arterial BP. BaPWV did not correlate with lung function parameters in the current study. Unlike ABI, which is an indicator of lumen stenosis and was positively correlated with pulmonary function, baPWV may be a better method for screening PAS in the general population. However, FEV1 and FVC were lower in the PAS group than in the non-PAS group. It has also been reported that airway limitations are associated with baPWV in a cigarette-smoking population, but not in a non-smoking population, which is consistent with the current study.

Ultrasound is a simple and non-invasive examination method that not only assesses morphological changes in vessels but can also evaluate blood vessel elasticity. This makes ultrasound a much more suitable method to assess vascular status in COPD patients. Detection of PAS using ultrasound revealed that the right brachial artery AccV (before and after the 6MWD test) and left dorsalis pedis artery AccV (after the 6MWD test) were significantly correlated with PAS occurrence in COPD patients in this study. Correlation analysis showed that AccV was positively correlated with baPWV in the bilateral brachial artery and left dorsalis pedis artery. This suggests that right brachial artery AccV may be used as an indicator of the degree of PAS in COPD patients, which aids the assessment of vascular disease risk in COPD patients. Since we are the first to use AccV to evaluate PAS, especially in high-risk COPD patients, the feasibility of this method needs to be further studied. A weak correlation between AccV and baPWV in the dorsalis pedis artery may be related to frequent obstruction and collateral circulation in the dorsalis pedis artery. Based on our findings, ultrasound could be an effective method to evaluate and screen for PAS in COPD patients.

## Conclusion

The prevalence of PAD in our study was lower in both COPD and non-COPD patients than previously reported. Patients with COPD had lower BP and poorer exercise tolerance than non-COPD patients. Detection of morphologic changes and right brachial artery AccV may be good methods to assess the degree of atherosclerosis. Further studies are needed to explore the feasibility of these methods.

### Limitations of this study

1. This study included only 112 patients in single-center outpatients;
2. Most patients with mild to moderate COPD;
3. This study is only a preliminary observational study, and we should continue to collect patients to evaluate the efficacy of the above indicators in the future.

## Data Availability

All data used during the study are available on reasonable request, Li Lin could be contacted if someone wants to request the data from this study.
